# Colchicine – an effective treatment for children with a clinical diagnosis of autoinflammatory diseases without pathogenic gene variants

**DOI:** 10.1186/s12969-021-00588-0

**Published:** 2021-09-14

**Authors:** Tatjana Welzel, Anna L. Wildermuth, Norbert Deschner, Susanne M. Benseler, Jasmin B. Kuemmerle-Deschner

**Affiliations:** 1 Pediatric Rheumatology and Autoinflammatory Reference Center, University Children’s Hospital Tuebingen, University of Tuebingen, Tuebingen, Germany; 2 Pediatric Pharmacology and Pharmacometrics, University Children’s Hospital Basel (UKBB), University of Basel, Basel, Switzerland; 3 Department of Anaesthesiology and Intensive Care Medicine, University Hospital Tuebingen, University of Tuebingen, Tuebingen, Germany; 4 Rheumatology, Department of Paediatrics, Alberta Children’s Hospital, Cumming School of Medicine, Alberta Children’s Hospital Research Institute, University of Calgary, Calgary, Alberta Canada

**Keywords:** Autoinflammatory diseases, Variants of unknown significance, Low penetrance variants, Effectiveness, Safety, Colchicine

## Abstract

**Background:**

Autoinflammatory diseases (AID) are rare chronic conditions with high disease burden, affecting children and adults. Clinically and genetically confirmed, AID can be effectively treated with targeted cytokine inhibition. In contrast, for patients with clinical AID symptoms without pathogenic gene variants, no treatment recommendations are available. Colchicine is approved and established as effective, safe and low-cost first-line therapy in Familial Mediterranean Fever. Up to now, efficacy data for colchicine in children with a clinical AID diagnosis without pathogenic gene variants are rare. This pilot study was performed to evaluate the effectiveness of colchicine in children with a clinical diagnosis of AID without pathogenic gene variants.

**Methods:**

A pilot cohort study of consecutive children with active clinical AID without pathogenic gene variants treated with colchicine monotherapy was performed between 01/2009 and 12/2018. Demographics, clinical and laboratory characteristics were determined serially. Colchicine dosing and safety were documented. Physician estimate of disease activity was captured on visual analogue scales (VAS). Primary outcome: Complete response (PGA ≤2 plus CRP ≤0.5 mg/dL and/or SAA ≤10 mg/L) at last follow-up. Secondary outcomes: partial/no response, flare characteristics and requirement for rescue therapies. Analysis: Nonparametric comparison of disease activity measures.

**Results:**

A total of 33 children were included; 39% were female. Median age at colchicine start was 3.8 years, median follow-up was 14.1 months. Clinical AID diagnoses included CAPS (24%), FMF (27%), PFAPA (43%) and unclassified AID (6%). At baseline, overall disease activity was moderate (PGA 4), inflammatory markers were elevated (CRP 12.1 mg/dL; SAA 289.2 mg/L), and 97% reported febrile flares. Outcome: 55% achieved complete response, 35% showed partial response and 58% had no febrile flares at last follow-up. Inflammatory markers (SAA: *p* < 0.0001, CRP: *p* < 0.005) and disease activity (*p* < 0.0001) decreased significantly. Overall, 93% of children experienced improvement of flare characteristics.

**Conclusion:**

Colchicine was found to be effective and safe in children with a clinical AID diagnosis in the absence of pathogenic gene variants. Colchicine is a low-cost treatment option for non-organ threatening AID.

## Background

Autoinflammatory diseases (AID) are rare conditions with high disease burden; many AID are caused by pathogenic gene variants leading to excessive production of pro-inflammatory cytokines [1, 2]. Typical AID symptoms include recurrent fevers, inflammation of joints, eyes, skin and serous membranes coupled with increased inflammatory markers [3]. However, the AID spectrum continuously expands and atypical presentations can result from somatic mosaicisms or low-penetrance variants [4, 5]. Genetic testing has been established in recent years, allowing a reliable and definitive diagnosis in patients with pathogenic gene variants together with clinical AID symptoms. For patients with clinical AID symptoms and negative or inconclusive genetic testing it remains still challenging to make a diagnosis.

Effective AID treatment is crucial to prevent morbidity, mortality, reduce health-related quality of life and high psychosocial burden. Clinically and genetically confirmed AID can be effectively treated with targeted cytokine inhibition [6]. In contrast, for patients with clinical AID symptoms without pathogenic gene variants or atypical AID symptoms, no treatment recommendations are available. This is particularly challenging for patients living in low-income countries.

Colchicine is approved and established as effective, safe and low-cost first-line therapy in Familial Mediterranean Fever (FMF) [7]. Furthermore, colchicine might be an effective treatment for children with clinical AID symptoms but negative or inconclusive genetic testing. In the past years several reports illustrated that colchicine might be beneficial in the therapeutic management of patients with other AID than FMF, particularly in unclassified AID [8–11]. However, more data on effectiveness and safety for colchicine in children with a clinical AID diagnosis without pathogenic gene variants are needed to support clinicians in their treatment decisions.

Therefore, the aims of this study were 1) to describe demographical, clinical and laboratory characteristics of children presenting with a clinical AID diagnosis without pathogenic gene variants and 2) to report the effectiveness of colchicine monotherapy in these patients.

## Patients and methods

A single-centre pilot cohort study of consecutive children with a clinical diagnosis of AID without likely pathogenic or pathogenic gene variants was performed between 01/2009 and 12/2018. Children and adolescents ≤18 years of age were included, if they had a clinical AID diagnosis after exclusion of other differential diagnoses based on standardized assessments of fever, constitutional symptoms and classical AID organ manifestations paired with elevated inflammatory markers in flares. The diagnosis was supported by diagnostic or classification criteria where available [12–14]. Patients not meeting a single specific set of AID criteria were considered unclassified AID. Furthermore, for study inclusion they had to fulfil the following criteria: 1) in the AID gene panel test (i) no detected gene variant including patients with periodic fever, aphthous stomatitis, pharyngitis and adenitis (PFAPA), or (ii) a gene variant of (likely) benign/uncertain significance according to the American College of Medical Genetics and Genomics [15], and 2) evidence of active disease. A genetic AID panel test for variants in the *MEFV, MVK, TNFRSF1A, NLRP3, NOD2, PSTPIP1* and *LPIN2* gene was performed in all patients in a certified laboratory. The AID gene panel was expended by several newly discovered genes associated with AID, such as *IL10RA, IL1RN, IL36RN, ADA2* during the study period. Children were excluded, if they had 1) evidence of amyloidosis or organ damage, 2) previous or ongoing biologic therapy, 3) met criteria for paediatric Behçet disease [16], 4) discontinued colchicine treatment or 5) were found to have significantly elevated liver enzymes (>3x upper limit of normal). Children with elevated liver enzymes were excluded from the study, because elevation of liver enzymes may be a side effect of colchicine therapy. Data was captured in the designated, institutional web-based Arthritis and Rheumatism Database and Information System (ARDIS) including standardized assessments of outcome measures at all visits [17]. Ethic approval was obtained from the University of Tuebingen Institutional Review Board (012/2017BO2). A waiver of patients informed consent for the study was obtained. The study was performed in compliance with the Helsinki Declaration.

### Demographics, clinical and laboratory features

Demographical data included clinical AID diagnosis, gender, self-reported ethnicity, median age at AID diagnosis and at colchicine treatment start. Furthermore, clinical symptoms and treatment at referral to the study center were analyzed. Flare frequency was defined in categories 0 to 4 with 0 = no flares, 1= > every six weeks, 2 = every five to six weeks, 3 = every three to four weeks and 4 = every one to two weeks; flare duration in days was defined in categories 0 to 3 with 0 = no flares, 1 = one to two days, 2 = three to four days and 3 = five or more days; fever during flares was defined as a body temperature ≥ 38°Celsius measured rectally, orally, axillary or at the ear. AID symptoms were captured using a symptom diary similar to the Autoinflammatory Disease Activity Index (AIDAI) [18]. At each visit, a complete physical examination and evaluation of possible AID complications were performed. The inflammatory markers C-reactive protein (CRP), serum amyloid A (SAA) and whole blood count, liver enzymes and kidney function tests were performed at each visit. Urine for proteinuria was analysed when urine sampling was possible at visit. The study defined three distinct time points: 1) baseline defined as time of colchicine treatment start, 2) first follow-up as visit after colchicine start and 3) last follow-up as last study visit.

### Colchicine therapy

Colchicine treatment was started at 0.5 to 1.0 mg/day. The therapeutic effect was monitored at three to six month visits. Based on disease activity and side effects colchicine dose was adjusted by 0.5 mg/day steps. During disease flares co-medication with non-steroidal anti-rheumatic drugs (NSAID) was allowed. Colchicine safety monitoring included gastrointestinal symptoms, whole blood count, liver and renal function. Colchicine was stopped if adverse events or intolerance occurred at the minimal dose of 0.5 mg/day. If the maximum tolerated colchicine dose did not result in improvement of disease activity anti-interleukin-1(IL-1) treatment was indicated.

### Definitions of disease activity and response

Disease activity was defined as physician global assessment (PGA) recorded on a 10 cm visual analogue scale (VAS) with 0 representing no disease activity and 10 maximum disease activity. Disease activity was categorized as mild (PGA ≤2), moderate (PGA > 2 ≤ 5) and high (PGA > 5). Laboratory evidence of disease activity included CRP > 0.5 mg/dL and/or SAA > 10 mg/L.

Colchicine response was defined as complete, partial and no response based on clinical disease activity and inflammatory markers. Response criteria were selected based on previous studies [19–22]. Complete response (CR) was defined as PGA ≤2 plus CRP ≤0.5 mg/dL and/or SAA ≤10 mg/L; partial response (PR) as PGA > 2 ≤ 5 plus CRP > 0.5 mg/dL ≤5 mg/dL and/or SAA > 10 mg/L ≤ 50 mg/L and no response (NR) as PGA > 5 and/or CRP > 5 mg/dL and/or SAA > 50 mg/L.

### Outcome

The primary outcome was CR to colchicine at last follow-up. Secondary outcomes included colchicine responses: 1) PR and NR at last follow-up, 2) CR, PR and NR at first follow-up; flare responses: 3) flare frequency, 4) flare duration, 5) fever during flares; colchicine impact of flares: 6) overall flare improvement defined as the composite of reduced flare frequency and shortening of flare duration and lower/no fever during flares, 7) any impact on flares defined as either reduced flare frequency or shortening of flare duration or lower/no fever during flare, 8) requirement for anti-IL-1 treatment and 9) colchicine safety.

### Analysis

Baseline demographics were analysed using descriptive statistics; median values and ranges, mean values and interquartile ranges were computed. Comparative analyses were conducted using parametric and nonparametric methods as appropriate. R (version 3.5.1; R Development Core Team, Vienna, Austria, (http://r-project.org)) was used for data analysis (CRP, PGA, SAA) and visual graphics.

## Results

### Patients

A total of 33 children were included in this pilot study. Of these, nine patients (27%) had a clinical diagnosis of FMF without *MEFV* variants. Eight patients (24%) were clinically diagnosed as Cryopyrin-associated periodic syndromes (CAPS) with low-penetrance *NLRP3* variants (*V198M, Q703K*) showing overlap characteristics for the mild to moderate CAPS phenotype. Typical CAPS symptoms were paired with atypical symptoms, such as abdominal pain, previously described for low-penetrance variants associated with CAPS phenotypes. PFAPA was diagnosed in 14 patients (43%). Two patients (6%) were diagnosed with an unclassified AID. Mediterranean ethnicity was reported by two thirds of patients (64%). A total of 13 patients were female (39%). The median age at AID diagnosis was 3.6 years (range 0.8–11.9) (Table 1). The clinical characteristics of the patients during flares are summarized in Table 2. An overview of previous treatment before referral to the study centre and change to colchicine is summarized in Table 3. All 33 children were treated with NSAIDs during flares.

**Table 1:** Demographic characteristics and follow-up time of children with a clinical AID diagnosis without pathogenic gene variants

	FMF ***N*** = 9	CAPS ***N*** = 8	PFAPA ***N*** = 14	Unclassified AID ***N*** = 2	Total cohort ***N*** = 33
Female gender, N (%)	4 (44)	3 (38)	6 (43)	0 (0)	13 (39)
Mediterranean ethnicity, N (%)	8 (89)	5 (63)	7 (50)	1 (50)	21 (64)
Age at AID diagnosis in years, median (range)	3.6 (0.9–7.8)	3.8 (0.8–6.1)	3.3 (0.9–8.5)	9.1 (6.2–11.9)	3.6 (0.8–11.9)
Age at colchicine start in years, median (range)	3.6 (1.2–10)	3.9 (0.8–7.5)	3.5 (1.2–9.1)	9.5 (6.3–12.6)	3.8 (0.8–12.6)
Age at last follow-up in years, median (range)	5.6 (2.5–11.6)	5.0 (1.7–9.7)	5.0 (2.3–10.6)	10.5 (6.9–14.1)	5.3 (1.7–14.1)
Time baseline to last follow-up in months, median (range)	15.2 (8.7–34.2)	11.2 (5.3–47.3)	13.7 (7.8–47.3)	12.7 (6.9–18.4)	14.1 (5.3–47.3)

*Abbreviations*: *AID* autoinflammatory disease, *FMF* Familial Mediterranean Fever, *CAPS* Cryopyrin-associated periodic syndromes, *PFAPA* periodic fever, aphthosis, pharyngitis and adenitis

**Table 2:** Clinical characteristics of children with a clinical AID diagnosis without pathogenic gene variants

	FMF ***N*** = 9	CAPS ***N*** = 8	PFAPA ***N*** = 14	Unclassified AID ***N*** = 2
Recurrent fever, N (%)	8 (89%)	8 (100)	14 (100)	2 (100)
Elevates CRP/SAA in flares, N (%)	9 (100)	8 (100)	14 (100)	2 (100)
Positive family history, N (%)	4 (44)	0	6 (43)	0
Rashes, urticaria like rashes, N (%)	0	6 (75)	0	0
Cold/stress induced flares, N (%)	0	3 (38)	0	1 (50)
Musculoskeletal pain, arthritis, N (%)	5 (55)	8 (100)	7 (50)	2 (100)
Skeletal abnormalities, N (%)	0	0	0	0
Breast pain, N (%)	0	0	0	0
Abdominal pain, N (%)	6 (67)	3 (38)	2 (14)	1 (50)
Diarrhoea, nausea, N (%)	0	3 (38)	0	0
Recurrent headaches, N (%)	0	1 (13)	0	1 (50)
Chronic aseptic meningitis, N (%)	0	0	0	0
Conjunctivitis, episcleritis, N (%)	0	2 (26)	0	0
Hearing loss, N (%)	0	0	0	0
Aphthous stomatitis, tonsillitis, pharyngitis, N (%)	0	0	14 (100)	0
Cervical lymphadenopathy; N (%)	0	0	13 (93)	0

*Abbreviations*: *AID* autoinflammatory disease, *FMF* Familial Mediterranean Fever, *CAPS* Cryopyrin-associated periodic syndromes, *PFAPA* periodic fever, aphthosis, pharyngitis and adenitis

**Table 3:** Treatment of children with a clinical AID diagnosis without pathogenic gene variants before colchicine start

	FMF ***N*** = 9	CAPS ***N*** = 8	PFAPA ***N*** = 14	Unclassified AID ***N*** = 2	Total cohort ***N*** = 33
Antibiotics, N (%)	1 (11)	3 (38)	3 (21)	0	7 (21)
Antihistamines N (%)	0	1 (13)	0	0	1 (3)
Corticosteroids, N (%)	0	3 (38)	7 (50)	1 (50)	11 (33)
NSAIDs, N (%)	9 (100)	8 (100)	14 (100)	2 (100)	33 (100)
Intravenous Ig’s N (%)	0	0	1 (7)	0	1 (3)
Tonsillectomy, N (%)	0	1 (13)	1 (7)	0	2 (6)

*Abbreviations*: *AID* autoinflammatory disease, *FMF* Familial Mediterranean Fever, *CAPS* Cryopyrin-associated periodic syndromes, *PFAPA* periodic fever, aphthous stomatitis, pharyngitis and adenitis, *NSAIDs* non-steroidal anti-rheumatic drugs, *Ig’s* Immunoglobulins

### Colchicine therapy

At baseline the median age of the cohort was 3.8 years (range 0.8–12.6). The median follow-up time at first follow-up was 6.4 months (range 2.1–9.2) and at last follow-up 14.1 months (range 5.3–47.3). At baseline, the majority of patients (91%) was started on a colchicine dose of 0.5 mg/day; only three patients (9%) received 1 mg/day. The median time for dose adjustment was 5.2 months (range 1.8 to 9 months). At first follow-up colchicine dose had to be adjusted in 18 patients (55%) including 13 patients in whom the colchicine dose was doubled (1 mg/day), three in whom it was tripled (1.5 mg/day), one patient in whom it was reduced (1 to 0.5 mg/day) and in one patient colchicine was discontinued. The mean colchicine dose was 0.8 mg/day at first follow-up. Dose was reduced in nine and increased in four patients between first and last follow-up. At last follow-up, 14 patients (44%) received 0.5 mg/day, 10 (31%) 1 mg/day and 8 (25%) 1.5 mg/day resulting in a mean colchicine dose of 0.9 mg/day for the whole study cohort.

### Disease activity and inflammatory markers

**Fig. 1:**
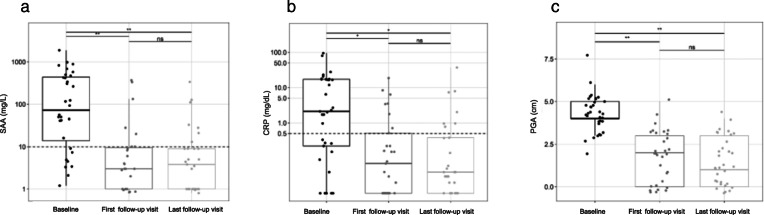
Disease activity and inflammatory markers in clinical AID without pathogenic gene variants treated with colchicine. Legend: **a)** Serum amyloid A (SAA), **b** C-reactive protein (CRP) and **c** disease activity are depicted at baseline, first follow-up and at last follow-up. The SAA and CRP values are presented on a log scale. SAA, CRP and the disease activity decreased significantly from baseline to first and last follow-up. There were no statistic significant changes (*p* > 0.05) between first and last follow-up. Significances were tested by Steel Dwass Methode. Abbreviations: *SAA* serum amyloid A, *CRP* C-reactive protein, *PGA* physician global assessment, *ns* not significant (*p* > 0.05), ** *p* < 0.0001, * *p* < 0.005

At baseline, the disease activity for the whole cohort was moderate with a median PGA of 4 (range 2–8). Two patients (6%) had high disease activity (PGA > 5); one showed mild disease activity (PGA ≤2). Disease activity decreased significantly for the whole cohort at first (*p* < 0.0001) and last follow-up (*p* < 0.0001) (Fig. 1, Table 4). At baseline, inflammatory markers were highly elevated with a mean CRP of 12.1 mg/dL (SD 21.28) and a mean SAA of 289.2 mg/L (SD 410.98). CRP and SAA values decreased to a mean CRP of 1.5 mg/dL (SD 3.85) and a mean SAA of 35.4 mg/L (SD 94.42) at first follow-up (CRP: *p* < 0.005; SAA: *p* < 0.0001) and to a mean CRP of 2 mg/dL (SD 7.01) and a mean SAA of 25.6 mg/L (SD 66.90) at last follow-up (CRP: *p* < 0.005; SAA: *p* < 0.0001) (Fig. 1, Table 4). No proteinuria was detected (Table 4).

**Table 4:** Disease activity and inflammatory markers in clinical AID without pathogenic gene variants treated with colchicine

	FMF ***N*** = 9	CAPS ***N*** = 8	PFAPA ***N*** = 14	Unclassified AID ***N*** = 2	Total cohort ***N*** = 33
**Disease activity (PGA) in cm (0 to 10 cm), median (range)**					
Baseline	4 (2–5)	4.5 (3–8)	4.5 (3–6)	3.5 (3–4)	4 (2–8)
First follow-up	2 (0–4)	2.5 (0–4)	2 (0–5)	1 (0–2)	2 (0–5)
Last follow-up	1 (0–3)	1.5 (0–3)	2 (0–4)	0.5 (0–1)	1 (0–4)
**CRP values in mg/dL (*N*** **< 0.5 mg/dL)**					
Baseline, mean ± SD (tested)	**18** ± 30.35 (9/9)	**14.7** ± 27.49 (8/8)	**8.5** ± 8.72 (14/14)	**0.05** ± 0.06 (2/2)	**12.1** ± 21.28 (33/33)
First follow-up, mean ± SD (tested)	**1.2** ± 2.15 (8/9)	**2** ± 3.38 (6/8)	**1.7** ± 5.15 (13/14)	**0.01** ± 0.00 (2/2)	**1.5** ± 3.85 (29/33)
Last follow-up, mean ± SD (tested)	**1.7** ± 3.13 (6/9)	**1** ± 2.55 (8/8)	**3** ± 10.22 (13/14)	**0.49** ± 0.67 (2/2)	**2** ± 7.01 (29/33)
**SAA values in mg/L (*N*** **< 10 mg/L)**					
Baseline, mean ± SD (tested)	**442.9** ± 668.45 (8/9)	**97.0** ± 117.22 (8/8)	**349.5** ± 320.26 (14/14)	**22.1** ± 28.21 (2/2)	**289.2** ± 410.98 (32/33)
First follow-up, mean ± SD (tested)	**66.8** ± 147.73 (6/9)	**6.7** ± 9.70 (7/8)	**39.1** ± 95.33 (13/14)	**0.8** ± 0 (1/2)	**35.4** ± 94.42 (27/33)
Last follow-up, mean ± SD (tested)	**6.7** ± 9.54 (7/9)	**51.7** ± 125.56 (7/8)	**16.9** ± 34.22 (13/14)	**56** ± 77.78 (2/2)	**25.6** ± 66.90 (29/33)
**Proteinuria, N (%)**					
Baseline (tested)	0 (0) (5/9)	0 (0) (3/8)	0 (0) (12/14)	0 (0) (2/2)	0 (0) (22/33)
First follow-up (tested)	0 (0) (6/9)	0 (0) (5/8)	0 (0) (10/14)	0 (0) (2/2)	0 (0) (23/33)
Last follow-up (tested)	0 (0) (7/9)	0 (0) (7/8)	0 (0) (11/14)	0 (0) (2/2)	0 (0) (27/33)

*Abbreviations*: *AID* autoinflammatory disease, *FMF* Familial Mediterranean Fever, *CAPS* Cryopyrin-associated periodic syndromes, *PFAPA* periodic fever, aphthosis, pharyngitis and adenitis, *w* weekly, *d* days, *CRP* C-reactive protein, *SAA* serum amyloid A, *mg* milligram, *dL* decilitre, *L* litre

### Outcome

#### Primary outcome

CR at last follow-up was achieved in 17/31 patients (55%).

#### Secondary Outcomes

**Fig. 2:**
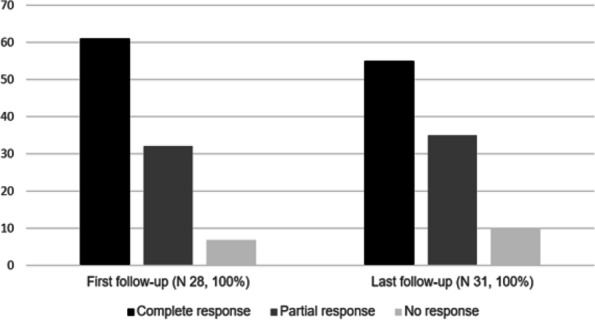
Colchicine response in children with a clinical AID diagnosis without pathogenic gene variants. Legend: Percentages of children with a clinical AID diagnosis without pathogenic gene variants with complete (PGA ≤2 cm plus CRP ≤0.5 mg/dL and/or SAA ≤10 mg/L), partial (PGA > 2 ≤ 5 cm plus CRP > 0.5 mg/dL ≤5 mg/dL and/or SAA > 10 mg/L ≤ 50 mg/L) and no (PGA > 5 and/or CRP > 5 mg/dL and/or SAA > 50 mg/L) colchicine response. Complete response was achieved in > 50% of patients, a third had a partial response and no response was achieved in ≤10%. Abbreviations: *N* patients number, *SAA* serum amyloid A, *CRP* C-reactive protein, *PGA* physician global assessment, *mg* milligram; *dL* decilitre; *L* litre

Colchicine responses: 1) PR at last follow-up was achieved by 11/31 patients (35%) and three patients (10%) showed NR. 2) At first follow-up, CR was achieved in 17/28 patients (61%), nine patients (32%) showed PR and two (7%) had NR (Fig. 2, Table 5).

**Table 5:** Colchicine outcome in children with a clinical AID diagnosis without pathogenic gene variants

	FMF ***N*** = 9	CAPS ***N*** = 8	PFAPA ***N*** = 14	unclassified AID ***N*** = 2
**Complete response** defined as PGA ≤2 cm plus CRP ≤0.5 mg/dL and/or SAA ≤10 mg/L, % (confidence interval)				
First follow-up, N (tested)	43 (*14.3; 76.4), 3 (7/9)*	43 (*14.3; 82.4), 3 (7/8)*	75 (*58.3; 100.0), 9 (12/14)*	100 (*100.0; 100.0), 2 (2/2)*
Last follow-up, N (tested)	63 (*37.5; 95.9), 5 (8/9)*	50 (*25.0; 89.2), 4 (8/8)*	54 (*30.8; 81.4), 7 (13/14)*	50 (*50.0; 100.0), 1 (2/2)*
**Partial response** defined as PGA > 2 ≤ 5 cm plus CRP > 0.5 mg/dL ≤5 mg/dL and/or SAA > 10 mg/L ≤ 50 mg/L, % (confidence interval)				
First follow-up, N (tested)	57 (28.6; 90.7), 4 (7/9)	43 (14.3; 82.4), 3 (7/8)	17 (0.0; 42.0), 2 (12/14)	0 (0.0; 85.6), 0 (2/2)
Last follow-up, N (tested)	37 (12.5; 70.9), 3 (8/9)	38 (12.5; 76.7), 3 (8/8)	31 (7.7; 58.3), 4 (13/14)	50 (50.0; 100.0), 1 (2/2)
**No response** defined as PGA > 5 and/or CRP > 5 mg/dL and/or SAA > 50 mg/L, % (confidence interval)				
First follow-up, N (tested)	0, 0 (7/9)	14 (0.0; 53.8), 1 (7/8)	8 (0.0; 33.6), 1 (12/14)	0, 0 (2/2)
Last follow-up, N (tested)	0, 0 (8/9)	12 (0.0; 51.7), 1 (8/8)	15 (0.0; 42.9), 2 (13/14)	0, 0 (2/2)
**Requirement of anti-IL-1 treatment,** N (%)				
First follow-up			1 (7)	
Last follow-up			1 (7)	
**Colchicine impact of flares**, N (%) reduced flare frequency or shorter flares or lower/no fever				
First follow-up, (tested)	7 (78), (9/9)	5 (71), (7/8)	12 (92), (13/14)	1 (50), (2/2)
Last follow-up, (tested)	9 (100), (9/9)	7 (100), (7/8)	10 (83), (12/14)	2 (100), (2/2)

*Abbreviations: AID* autoinflammatory disease, *FMF* Familial Mediterranean Fever, *CAPS* Cryopyrin-associated periodic syndromes, *PFAPA* periodic fever, aphthosis, pharyngitis and adenitis, *PGA* Physician global assessment, *CI* confidence interval (95 CL lower; upper), *cm* centimetre, *CRP* c reactive protein, *SAA* serum amyloid A, *mg* milligram, *dL* decilitre, *L* litre

Flare responses: 3) Flare frequency improved in 22/31 patients (71%) with available flare frequency data throughout the study. At baseline, nine patients (28%) reported flares every one to two weeks, 11 (34%) had flares every three to four weeks, four (13%) had flares every five to six weeks and eight (25%) had a flare frequency of > every six weeks. At baseline, median flare frequency category was 3 (range 1–4). At first follow-up, the median flare frequency category decreased to 1 (range 0–3). No flares were reported by 13/31 patients (42%). At last follow-up, no flares were reported by 16 patients (52%). The median flare frequency decreased to 0 (range 0–3). 4) Flare duration was reduced in 23/29 patients (79%) with available flare duration data at last follow-up. At baseline, 20/31 patients (65%) reported a flare lasting ≥ five days, 10 (32%) three to four days and one (3%) had a flare duration of one to two days. At baseline, the median category of the flare duration was 3, (range 1–3) and at first follow-up, it decreased to 2 (range 0–3). At last follow-up, the median category of flare duration was 0 (range 0–3). Consequently, at last follow-up 5/29 patients (17%) reported a flare duration of ≥ five days, 6 (21%) reported three to four days, three (10%) one to two days and 15 patients (52%) reported no flares (Table 6). 5) At baseline, 32 patients (97%) reported febrile flares. Of these, three patients had body temperatures between 38.0 and 38.4 °Celsius and 29 patients reported body temperatures ranging between 38.5 and 41.1°Celsius. At first follow-up, febrile flares were reported by 17/31 patients (55%). Of these, two patients had body temperatures ranging between 38.0 and 38.4° Celsius and all others had body temperatures varying between 38.5 and 41.0° Celsius. At last follow-up, 13/31 patients (42%) had febrile flares (Table 6) with body temperatures ranging between 39.0 and 40.3° Celsius.

**Table 6:** Flare response of children with a clinical AID diagnosis without pathogenic gene variants and colchicine

	FMF ***N*** = 9	CAPS ***N*** = 8	PFAPA ***N*** = 14	unclassified AID ***N*** = 2	Total cohort ***N*** = 33
**Flare frequencies** in categories: 0: no flare, 1: >every 6 weeks, 2: every 5–6 weeks, 3: every 3–4 weeks, 4: every 1–2 weeks, median (range)					
Baseline	2 (1–4)	3 (1–4)	3 (1–4)	2 (1–3)	3 (1–4)
First follow-up	1 (0–3)	1 (0–3)	0 (0–3)	1 (0–1)	1 (0–3)
Last follow-up	0 (0–3)	1 (0–3)	1 (0–3)	1 (0–1)	0 (0–3)
**Median flare duration** in category: 0: no flare, 1: 1–2 days, 2: 3–4 days, 3: ≥5 days, median (range)					
Baseline	2 (1–3)	3 (3)	3 (2–3)	3 (3)	3 (1–3)
First follow-up	2 (0–3)	2 (0–3)	0 (0–3)	2 (0–3)	2 (0–3)
Last follow-up	0 (0–2)	0 (0–3)	2 (0–3)	1 (0–1)	0 (0–3)
**Patients with febrile flares,** defined as ≥38.0° Celsius body temperature (orally, rectally, axillary, ear) during last flare, N (%)					
Baseline, (tested)	8 (89), (9/9)	8 (100), (8/8)	14 (100), (14/14)	2 (100), (2/2)	32 (97), (33/33)
First follow-up, (tested)	6 (67), (9/9)	5 (63), (8/8)	5 (42), (12/14)	1 (50), (2/2)	17 (55), (31/33)
Last follow-up, (tested)	2 (22), (9/9)	4 (50), (8/8)	6 (50), (12/14)	1 (50), (2/2)	13 (42), (31/33)

*Abbreviations*: *AID* autoinflammatory disease, *FMF* Familial Mediterranean Fever, *CAPS* Cryopyrin-associated periodic syndromes, *PFAPA* periodic fever, aphthosis, pharyngitis and adenitis

Colchicine impact on flares: 6) Overall improvement of flares was reported by 12/27 patients (44%) with available flare data at first follow-up and by 16/29 patients (55%) at last follow-up. 7) At first follow-up, 81% documented an improvement in at least one flare characteristic; the number increased to 93% of patients at last follow-up.

Requirement of anti-IL-1 treatment: 8) One PFAPA patient (3%) discontinued colchicine and started anti-IL-1 treatment between baseline and first follow-up, due to colchicine failure. This was the only patient who required anti-IL-treatment at first and last follow-up (Table 5).

Safety: 9) Colchicine dose adjustments were required in five patients (15%) most commonly due to gastrointestinal symptoms of abdominal pain, diarrhoea and nausea. Dose reduction from 1 mg to 0.5 mg/day resulted in resolution of gastrointestinal symptoms in four patients, one reported intermittent mild abdominal pain. No colchicine related safety signals were identified and no colchicine discontinuation was necessary.

## Discussion

This is the first study to systematically evaluate colchicine monotherapy for children with a clinical diagnosis of AID without pathogenic gene variants including children with a clinical diagnosis of FMF, CAPS, PFAPA and those with clinically unclassified AID. Colchicine was found to be effective and safe. At baseline, children displayed moderate disease activity (PGA 4) and elevated inflammatory markers (CRP 12.1 mg/dL; SAA 289.2 mg/L); frequent, severe febrile flares were documented in 97% of the patients. More than half of the patients experienced CR to colchicine at last follow-up including minimal to no disease activity and normalized inflammatory markers. An additional 35% of children had a PR. Overall, the study documented a significant decrease in disease activity (*p* < 0.0001) and decrease of inflammatory markers (SAA: *p* < 0.0001, CRP: *p* < 0.005) due to colchicine. In 93% of patients flares improved and 58% reported no febrile flares at last follow-up. These data suggest that colchicine should be considered for children with a clinical diagnosis of AID in the absence of pathogenic gene variants.

Colchicine was effective across different AID in reducing clinical and laboratory disease activity. Overall, high response rates were documented for FMF (100%), CAPS (88%), PFAPA (85%) and unclassified AID (100%).

In this pilot study, colchicine controlled disease activity in PFAPA patients and had an excellent effect on flare prevention. A total of 83% reported a positive impact of flare characteristics at last follow-up. Importantly, 75% of PFAPA patients reported less frequent flares. In 2008, Tasher et al. reported that eight out of nine PFAPA patients (89%) treated with colchicine had a significantly decreased flare frequency [23]. Similarly, Butbul Aviel et al. observed significantly reduced flares in eight children with PFAPA treated with colchicine compared to pre-treatment and to ten untreated controls [24]. These findings support our results, highlighting that colchicine is particularly effective in preventing PFAPA flares. However, more studies including larger cohorts of PFAPA patients are needed, to confirm these findings.

Colchicine was effective in children with CAPS and low-penetrance *NLRP3* variants. All included patients were symptomatic requiring treatment. Patients with low-penetrance *NLRP3* variants were previously shown to be at low risk for severe organ damage [5, 25, 26]. Overall, 88% of CAPS patients showed a colchicine response (four CR, three PR). Importantly, all patients (100%) reported a positive impact on disease flares resulting in less frequent flares and shorted flare duration. To date, colchicine therapy has not been systematically studied for CAPS and low-penetrance variants. A recent case report documented colchicine effectiveness in an elderly women with cold-induced urticarial-like rush and a pathogenic *NLRP3* variant (A439V) and an additional *MEFV* variant (E148Q) [27]. Among 94 CAPS patients analysed in the Eurofever Registry none was treated with colchicine [28]. To date, treatment recommendations include anti-IL-1 treatment for the whole spectrum of CAPS [6]. The results of this pilot study suggest that colchicine should be considered in the proposed CAPS treat-to-target approach, which is anchored in the presenting disease phenotype, disease activity and risk of organ damage [29]. CAPS patients with mild to moderate disease activity and low risk of organ damage may benefit from colchicine monotherapy as first-line agent. In addition, the low-cost colchicine therapy may provide an important option for low-income countries.

Colchicine was effective in patients with unclassified AID. All patients reported a positive impact on flares, there were no non-responders. Similarly, Papa et al., reported a positive colchicine effect in 78% variant-negative patients with undifferentiated recurrent fevers [10]. In addition, Chandrakasan et al. demonstrated a positive effect of colchicine in 15 patients with clinical periodic fever syndromes and negative or inconclusive genetics [11].

Not surprisingly, colchicine was effective in the studied children with FMF. All pathogenic gene variant-negative FMF patients showed colchicine response (63% CR, 37% PR). A positive effect on flares was reported by 100%. Colchicine is an established first-line therapy in FMF, preventing and aborting flares effectively [7, 30, 31]. Among 121 FMF patients analysed in the Eurofever Registry 79% had two *MEFV* variants, 17% had one *MEFV* variant, three patients had no variants and two were not tested [28]. All received colchicine and 62% achieved CR and 36% PR whereas two failed to respond [28]. Colchicine effectiveness in controlling disease activity is observed in variant-negative and variant-positive FMF patients [32]. These findings support our data that variant-negative FMF patients have good colchicine response rates.

Colchicine doses of 0.5 to 1 mg/day were effective and safe in the majority of children with clinically diagnosed AID without pathogenic gene variants. A total of 91% of the patients were commenced on 0.5 mg and 9% on 1 mg of colchicine daily. At first follow-up a significant reduction of inflammatory markers and disease activity was documented. Dose adjustments were made to optimize effectiveness and safety. At last follow-up, 44% of patients received 0.5 mg/day, 31% 1 mg/day and 25% 1.5 mg/day; in five children a dose reduction from 1 to 0.5 mg/day due to gastrointestinal side effects was required.

All PFAPA patients in this study cohort were commenced on 0.5 mg/day colchicine at baseline. At last follow-up, more than half of children had required dose adjustment with 31% of patients receiving 1 mg/day and 23% 1.5 mg/day. Recommended daily colchicine dose ranges between 0.5 to 1 mg for flare prevention in PFAPA and should be considered in patients, in whom corticosteroids have resulted in an increased flare frequency [33, 34]. Recently, the CARRA PFAPA working group has published consensus treatment plans, which should be evaluated in future pilot studies [35]. For the prophylaxis arm colchicine at 0.5 to 1.25 mg/day was recommended [35].

The majority of CAPS patients in this pilot study was started on colchicine 0.5 mg/day, only one received 1 mg/day. At last follow-up, 38% were treated with 0.5 mg, 38% with 1 mg and 24% with 1.5 mg/day. One child with unclassified AID was started on 0.5 mg/day of colchicine the other on 1 mg/day. At last follow-up both received 0.5 mg/day of colchicine. Currently, there is a complete lack of colchicine dosing recommendations for CAPS and for unclassified AID.

Most FMF patients were started on 0.5 mg/day of colchicine; one received 1 mg/day. At last follow-up, a third remained on 0.5 mg/day, another third received 1 mg/day and 1.5 mg/day respectively; none required dose increase to 2 mg/day. FMF treatment recommendations suggest age adjusted colchicine dosing regimens: for children < 5 years a starting dose of ≤0.5 mg/day (or ≤ 0.6 mg/day when the only available tablets contain 0.6 mg) and for 5–10 years starting doses of 0.5–1.0 mg/day (or 1.2 mg/day) [7, 30]. Children > 10 years and adults should be started at colchicine doses of 1.0–1.5 mg/day (or 1.8 mg/day) [7, 30]. The maximum daily colchicine dose should not exceed 2 mg in children and 3 mg in adults [7, 30]. An estimated 5 to 10% of colchicine treated patients may experience side effects such as diarrhoea, vomiting and nausea [36]. Dose adjustments may be necessary to reduce individual toxicity. Furthermore, dosing requirements may be related to the genotype [32, 37]. Ben-Zvi et al. suggested that control of disease activity in gene variant-negative FMF patients can be achieved with significantly lower colchicine doses compared to homozygous variant-positive FMF patients (M694V) [32]. Knieper et al. showed that there were no significant colchicine average dose differences between homozygous and compound heterozygous variant-positive FMF patients (M694V; M680I; M694V/M680I; M694V/V726A) [37]. In contrast, patients with the M694V/E148Q genotype or any heterozygous variant had significantly lower average colchicine doses [37]. Dose increases may be beneficial in variant-negative FMF patients and may result in reduced flare frequency, in particular when rather low colchicine doses are increased [37].

In this study the starting dose of colchicine varied between 0.5 mg/day (91%) and 1 mg/day (9%) resulting in a significant decrease in disease activity and inflammatory markers at first follow-up. This data suggests to start colchicine in children with a clinical diagnosis of AID without pathogenic gene variants irrespective of age with 0.5 mg/day. Dose adjustments might be required over time.

The study has several limitations. The sample size was small. However, the study aimed to include a very specific group of children with clinically diagnosed AID without pathogenic gene variants. Tuebingen is a reference center particularly providing support for AID patients with high disease activity, comorbidities, need of biologic therapy and unclear phenotype. The reference center takes care for PFAPA patients with high disease activity, non-response to corticosteroids on demand, unclear phenotype or suspicion of an overlap to another AID. Therefore, the PFAPA patient cohort is rather small compared to other AID at the centre. Furthermore, a comprehensive clinical and genetic work-up, including advanced genetic and functional testing helps to make a clinical and genetic AID diagnosis in several previously undiagnosed children with AID characteristics. Consequently, only few patients fulfilled the very strict inclusion criteria of no ongoing/previous biologic therapy, no organ damage, and no genetically confirmed AID diagnosis. Moreover, there was missing data as a real life cohort was studied. However, standardized assessment of the included patients by using the AIDAI and advanced laboratory testing combined with standardized outcome evaluation resulted in comparable high-quality data captured in the ARDIS.

## Conclusion

This study suggests that colchicine is an effective and safe therapy in children with a clinical diagnosis of AID without pathogenic gene variants. Colchicine may be considered as an effective first-line therapy in children with moderate to low disease activity and low risk of organ damage. Colchicine can result in a significant decrease in systemic inflammation, reduction of disease activity, decrease of flare frequency and shortening of flare duration. Therefore, colchicine is a low-cost, safe and effective treatment approach enabling reduction of morbidity and mortality.

## Data Availability

The dataset generated and analyzed during this study is not publicly available, but is available from the corresponding author on reasonable request after obtaining an ethical approval.

## Abbreviation

AID: Autoinflammatory diseases
AIDAI: Autoinflammatory Disease Activity Index
ARDIS: Arthritis and Rheumatism Database and Information System
CAPS: Cryopyrin-associated periodic syndromes
CR: Complete response
CRP: C-reactive protein
FMF: Familial Mediterranean Fever
IL-1: Interleukin-1
NR: No response
PFAPA: Periodic fever, aphthosis, pharyngitis and adenitis
PGA: Physician global assessment
PR: Partial response
SAA: Serum amyloid A
VAS: Visual analogue scale
